# New records, detailed distribution and abundance of rove-beetles (Insecta, Coleoptera, Staphylinidae) collected between 1990 and 2015 in Azores (Portugal) with an updated checklist

**DOI:** 10.3897/BDJ.10.e78896

**Published:** 2022-02-24

**Authors:** Paulo A. V. Borges, Lucas Lamelas-Lopez, Volker Assing, Michael Schülke

**Affiliations:** 1 cE3c – Centre for Ecology, Evolution and Environmental Changes / Azorean Biodiversity Group and Universidade dos Açores, Rua Capitão João d’Ávila, São Pedro, 9700-042, Angra do Heroísmo, Azores, Portugal cE3c – Centre for Ecology, Evolution and Environmental Changes / Azorean Biodiversity Group and Universidade dos Açores, Rua Capitão João d’Ávila, São Pedro, 9700-042 Angra do Heroísmo, Azores Portugal; 2 Gabelsbergerstraße 2, 30163, Hannover, Germany Gabelsbergerstraße 2, 30163 Hannover Germany; 3 Blankenfelder Straße 99, D-13127, Berlin, Germany Blankenfelder Straße 99, D-13127 Berlin Germany

**Keywords:** arthropods, Staphylinidae, Coleoptera, Azorean native forest, biodiversity, dataset, inventory, long term monitoring

## Abstract

**Background:**

The dataset we present consists of an inventory compiling all records and knowledge about Staphylinidae (Insecta, Coleoptera) in the Azores and is part of a long-term monitoring performed between 1990 and 2015 in different habitat types of eight islands of the Azores Archipelago. Most samples come from the BALA project (Biodiversity of Arthropods from the Laurisilva of Azores) that sampled native forests in the Azores. Additional sampled habitats include exotic forests, intensive and semi-natural pasturelands, orchards, caves and lava flows. Most of the records (about 96.7%) were collected in standardised sampling campaigns, which included pitfall traps and beating transect protocols. Non-standardised records are based on hand-collecting and sifting, as well as cave, colour and malaise traps.

**New information:**

We provide a long-term inventory of Staphylinidae (Insecta, Coleoptera) collected in the course of several standardised sampling campaigns and recorded with non-standardised methods. We collected a total of 10744 specimens belonging to 69 identified species of Staphylinidae, which represents 51% of the species known from the Azores Archipelago. Four endemic species were sampled, representing 40% of the known Azorean endemic species. From this dataset, seven species are new for the Azores: *Aleocharafunebris* Wollaston, 1864; *Amischaforcipata* Mulsant & Rey, 1873; *Blediusunicornis* (Germar, 1825); *Carpelimustroglodytes* (Erichson, 1840); *Cyphaseminulum* (Erichson, 1839); *Paraphloeostibagayndahensis* (MacLeay, 1871); *Tachyporuscaucasicus* Kolenati, 1846. We also registered a total of 66 new island records for eight Azorean islands. This contribution continues a series of publications on the distribution and abundance of Azorean arthropods. We also provide an updated list of Azorean rove-beetles (Staphylinidae) that now includes 136 species, ten of them considered Azorean endemics.

## Introduction

According to the latest list of rove-beetles from the Azores (Assing 2010), a total of 115 species and subspecies of Staphylinidae is known from the Azores, which is 21% of the known beetle fauna of the Archipelago (total number of beetle species in the Azores: 536 species and subspecies; see Oromí et al. 2010). Being one of the most diverse families of beetles, this group of mostly predatory insects is also known for its taxonomic complexity and frequent erroneous identifications (Barratt et al. 2003, Shaw and Solodovnikov 2016).

Despite the high species richness, very few endemic species have been recorded from the Archipelago; only ten species are confirmed as endemic in this paper (see Suppl. material 1). Several endemic staphylinids were discovered during the first scientific expeditions to the Azores (Crotch 1867, Fauvel 1900, Bernhauer 1936, Bernhauer 1940), others were found only recently (Israelson 1985, Pace 2004, Assing 2013). In addition to these works, the main contributors to the study of Azorean Staphylinidae were: Fauvel (1897), Fauvel (1898), Fauvel (1902), Méquignon (1942), Jarrige (1953), Smetana (1970), Brinck (1977), Serrano (1982), Israelson (1984), Gillerfors (1986), Gillerfors (1988), Serrano and Borges (1987), Borges and Serrano (1989) and Borges (1990), who added new records and provided taxonomic information.

However, in contrast to other Macaronesian archipelagos (see, for example, Assing and Schülke 2006), a recent revision of the Staphylinidae fauna of the Azores has not been attempted.

The main objective of this contribution is to provide an inventory of the Staphylinidae (Coleoptera, Insecta), based on a compilation of records from long-term monitoring sampling campaigns performed in eight islands of Azores between 1990 and 2015. This contribution is part of a series of publications on the distribution and abundance of Azorean arthropods (Borges et al. 2016, Borges et al. 2017).

## General description

### Purpose

The main objective is to provide an inventory of the Staphylinidae (Coleoptera, Insecta), based on a compilation of records from long-term monitoring sampling campaigns performed in eight islands of Azores between 1990 and 2015. In addition, an updated checklist of Azorean Staphylinidae is provided.

### Additional information

This contribution is part of a series of publications on the distribution and abundance of Azorean arthropods (Borges et al. 2016, Borges et al. 2017) and updates the latest checklist of Azorean Staphylinidae (see Assing 2010) .

Most data come from the following studies (see also Suppl. material 1): “1” – Confirmed occurrence, (see Assing 2010 list); BALA - records based on BALA protocol (see Borges et al. 2016; samples part of current study); LAND-USE (see Cardoso et al. 2009, samples part of current study); MACDIV (see Malumbres-Olarte et al. 2019); ASSING – Non-standardised samples of one of us (Volker Assing) (part of current study); Marcelino - Marcelino et al. (2021); INTERF (data from project INTERFRUTA part of current study); WET – from Borges et al. (2018); AGRO – see (Borges et al. 2021b); SLAM - Long term monitoring of Azorean forests using SLAM traps for Terceira Island (see Matthews et al. 2018, Borges et al. 2020, Tsafack et al. 2021); SLAM for other islands (unpublished data); ASSING 2013 (new species described in Assing 2013).

In this same Checklist of Azorean Staphylinidae (Suppl. material 1), we provide some relevant information related to taxonomic or nomenclature changes, splitting it into four columns “synonym”, “different combination”, “misidentification” and “emendation/misspelling”.

## Project description

### Title

Inventory of the Azorean rove-beetles (Insecta, Coleoptera, Staphylinidae)

### Personnel

Leader: Paulo A. V. Borges,

Fieldwork BALA project (Gaspar et al. 2008, Borges et al. 2016): Ana Santos, Álvaro Vitorino, Ana Rodrigues, Anabela Arraiol, Annabella Borges, Artur Serrano, Carla Rego, Carlos Aguiar, Catarina Melo, Clara Gaspar, Emanuel Barcelos, Fernando Pereira, Francisco Dinis, François Rigal, Genage André, Hugo Mas, Isabel R. Amorim, João Amaral, João Moniz, Joaquín Hortal, Kostas Triantis, Lara Dinis, Luís Vieira, Paula Gonçalves, Pedro Cardoso, Sandra Jarroca, Sérvio Ribeiro.

Fieldwork for SLAM - Long term monitoring of Azorean forests using SLAM traps; see list at Costa and Borges (2021).

Fieldwork for LAND-USE project: see list at Cardoso et al. (2009).

Fieldwork for MACDIV: see list at Malumbres-Olarte et al. (2019).

Taxonomists: Michael Schülke, Paulo A. V. Borges, Volker Assing.

### Study area description

**Figure 1.**
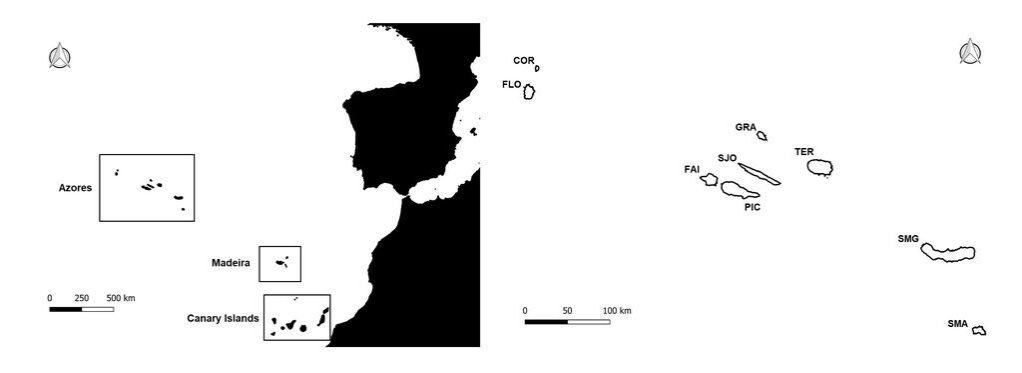
The Azores Archipelago with its location in the middle Atlantic (left panel) and the nine Azorean islands (right panel): the western group (COR - Corvo and FLO - Flores), the central group (FAI - Faial, PIC - Pico, GRA - Graciosa, SJG - São Jorge and TER - Terceira) and the eastern group (SMG - São Miguel and SMA - Santa Maria).

The Azores Archipelago extends for 615 km and is located in the North Atlantic Ocean (37-40°N, 25-31°W), about 1600 km from Europe and 2200 km from North America (Fig. 1). The Archipelago is formed by nine main islands and some small islets, all of them of volcanic origin. The islands are divided into three main groups: the western group (Corvo and Flores), the central group (Faial, Pico, Graciosa, São Jorge and Terceira) and the eastern group (São Miguel and Santa Maria). The climate is temperate oceanic, with regular and abundant rainfall, high levels of relative humidity, above 95% on average in native forests and persistent winds, mainly during the winter and autumn seasons. Since Portuguese colonisation in the 15^th^ century, the landscape of the Azores has been dramatically altered by replacing native forests with forest tree plantations, crops, pastures and urban areas. Currently, the native laurel forest comprises about 5% of the total surface of the Archipelago and has remained only at higher elevations and in inaccessible areas of the islands (Gaspar et al. 2008, Borges et al. 2020).

### Design description

This inventory of Staphylinidae includes records of several standardised sampling campaigns and non-standardised observations, performed between 1990 and 2015 (Gaspar et al. 2008, Borges et al. 2016). Most of the records (about 96.7%) were collected in standardised sampling campaigns, which included pitfall and beating protocols. Non-standardised records are based on hand-collecting and sifting, as well as cave, colour and malaise traps. Additional non-standard records are based on cave, colour and malaise traps and collecting trips conducted by Volker Assing in Terceira, São Miguel and Santa Maria islands in 2013 and by Andreas Kleeberg in Pico and São Miguel in 2015. Collected samples were sorted and subsequently identified by an expert taxonomist in laboratory.

### Funding

Species collecting was funded mostly by four projects:

“Reservas Florestais dos Açores: Cartografia e Inventariação dos Artrópodes Endémicos dos Açores” (BALA) (Direcção Regional dos Recursos Florestais, project 17.01-080203) (1999-2003);

“Agriculture, habitat fragmentation, indicator species and conservation of endemic fauna and flora in the Azores – the 2010 Target” (Direcção Regional da Ciência e Tecnologia, DRCT - Postdoc M112/F/014/2007) (2007-2009);

"Predicting extinctions on islands: a multi-scale assessment” (Fundação para a Ciência e Tecnologia- FCT- PTDC/BIA-BEC/100182/2008) (2010-2013);

“Understanding biodiversity dynamics in tropical and subtropical islands as an aid to science based conservation action” (ISLANDBIODIV) (Fundação para a Ciência e Tecnologia , FCT/NETBIOME/0003).

The database management was funded by FEDER (85%) and by Azorean Public funds (15%) through Operational Programme Azores 2020, under the project AZORESBIOPORTAL –PORBIOTA (ACORES-01-0145-FEDER-000072).

## Sampling methods

### Study extent

The study was conducted in several habitats of eight islands of the Azores Archipelago (Fig. 1): Flores, São Jorge, Pico, Faial, Terceira, Graciosa, São Miguel and Santa Maria Islands. The sampled habitats include exotic, mixed and native forests, intensive and semi-natural pastures, orchards, caves and lava flows (see details in Event Table at Borges et al. 2021a).

### Sampling description

This inventory of Staphylinidae (Coleoptera) includes records obtained in several standardised sampling campaigns and by non-standardised methods performed between 1990 and 2015. Most of the records (96.7%) are based on standardised sampling campaigns, which included pitfall and beating protocols. The used methodology is in accordance with BALA protocol - Biodiversity of Arthropods of the Laurissilva of Azores (Gaspar et al. 2008,Borges et al. 2016) implemented during BALA I (1999-2004) and BALA II (2010-2011) projects. Non-standardised records are based on hand-collecting and sifting, as well as cave, colour and malaise traps.

Standardised sampling campaigns included pitfall traps and beating transects. Pitfall trap transect protocols were conducted with 33 cl plastic cups, partially filled with propylene glycol, in the soil (cup rim at surface level) every 5 m. Traps were protected from rain using a plastic plate, placed about 5 cm above surface level and fixed to the ground with wire. The pitfall traps remained active in the field for 14 days. Beating transects were performed by beating the canopy of woody vegetation, using a beating tray. The protocol was conducted when the vegetation was dry. A 5 m wide square was established every 15 m (total of 10 squares per transect). Two woody plant specimens of the most abundant species (up to three species when available) were sampled in each square. For each selected plant, a branch was chosen at random and a beating tray placed beneath it. The tray consisted of a 1 m wide and 60 cm deep cloth inverted pyramid, with a plastic bag at the vertex. Five beatings were made using a stick for each plant individual sampled.

### Quality control

All sorted specimens were identified by a taxonomical expert. Taxonomic nomenclature followed: Schülke and Smetana (2015), Brunke et al. (2021), Yoo et al. (2021).

In terms of species colonisation status, we followed two classifications:

1) For the GBIF database that incoporates occurrence data, we followed the information in the last checklist of Azorean Staphylinidae (Assing 2010). This information is analysed through the main text of this manuscript.

2) However, species classified as "native" by Volker Assing (Assing 2010) have a dubious colonisation origin and were probably inadvertently introduced from the Western Palaearctic. It is highly probable that this happened following human colonisation of the islands and prior to the first reports on Azorean beetles in the 19^th^ century. Exceptions are *Athetapasadenae* and *Hypomedondebilicornis*, for which the native distribution is unknown.

For that reason, in the current checklist of Azorean Staphylinidae (Suppl. material 1), we now add a column in which we create a new possible tentative categorisation for the colonisation status of the species as follows:

- endemic: species for which we have some evidence that they are true endemics, occurring mostly in native habitats.

- Doubtfully endemic: species whose status is doubtful, based on our current knowledge on the distribution of congeneric species.

- Non-endemic: these include all the previous named as “native” and “introduced” since, in most cases, we currently have no confidence on their status.

- Non-endemic cosmopolitan: these include species with cosmopolitan distribution.

## Geographic coverage

### Description

Azores, Portugal (Flores, São Jorge, Pico, Faial, Terceira, Graciosa, São Miguel and Santa Maria).

### Coordinates

36.862 and 39.623 Latitude; -31.399 and -24.895 Longitude.

## Taxonomic coverage

### Description

This publication covers the Staphylinidae family (Insecta, Coleoptera).

### Taxa included

**Table:** 

Rank	Scientific Name	Common Name
family	Staphylinidae	Rove-beetles

## Traits coverage

Not available

## Temporal coverage

**Data range:** 1990-6-01 – 2015-9-30.

## Collection data

### Collection name

Entomoteca Dalberto Teixeira Pombo

### Collection identifier

DTP

### Specimen preservation method

Ethanol 96% for posterior DNA analyses

### Curatorial unit

Curator: Paulo A. V. Borges

## Usage licence

### Usage licence

Creative Commons Public Domain Waiver (CC-Zero)

## Data resources

### Data package title

Inventory of the Azorean rove-beetles (Insecta, Coleoptera, Staphylinidae)

### Resource link

https://www.gbif.org/dataset/81df7e48-1f76-4125-901b-140bd96dfa49

### Alternative identifiers

http://ipt.gbif.pt/ipt/resource?r=staphylinidae_occurrences_azores

### Number of data sets

2

### Data set 1.

#### Data set name

Event Table

#### Data format

Darwin Core Archive format

#### Number of columns

22

#### Character set

UTF-8

#### Download URL

http://ipt.gbif.pt/ipt/resource?r=staphylinidae_occurrences_azores

#### Data format version

Version 1.5

#### Description

The dataset was published in Global Biodiversity Information Facility platform, GBIF (Borges et al. 2021a). The following data table includes all the records for which a taxonomic identification of the species was possible. The dataset submitted to GBIF is structured as a sample event dataset that has been published as a Darwin Core Archive (DwCA), which is a standardised format for sharing biodiversity data as a set of one or more data tables. The core data file contains 2731 records (eventID). This IPT (Integrated Publishing Toolkit) archives the data and thus serves as the data repository. The data and resource metadata are available for download in the Portuguese GBIF Portal IPT (Borges et al. 2021a).

**Data set 1.** 

Column label	Column description
eventID	Identifier of the events, unique for the dataset.
stateProvince	Name of the region of the sampling site.
islandGroup	Name of archipelago.
island	Name of the island.
country	Country of the sampling site.
countryCode	ISO code of the country of the sampling site.
municipality	Municipality of the sampling site.
decimalLongitude	Approximate centre point decimal longitude of the field site in GPS coordinates.
decimalLatitude	Approximate centre point decimal latitude of the field site in GPS coordinates.
geodeticDatum	The ellipsoid, geodetic datum or spatial reference system (SRS) upon which the geographic coordinates given in decimalLatitude and decimalLongitude are based.
coordinateUncertaintyInMetres	Uncertainty of the coordinates of the centre of the sampling plot in metres.
coordinatePrecision	A decimal representation of the precision of the coordinates given in the decimalLatitude and decimalLongitude.
georeferenceSources	A list (concatenated and separated) of maps, gazetteers or other resources used to georeference the Location, described specifically enough to allow anyone in the future to use the same resources.
locationID	Identifier of the location.
fieldNumber	Code of the sample.
locationRemarks	Comments or notes about the Location.
locality	Name of the locality.
habitat	The habitat of the sample.
year	Year of the event.
month	Month of the event.
eventDate	Date or date range the record was collected.
samplingProtocol	The sampling protocol used to capture the species.

### Data set 2.

#### Data set name

Occurrence Table

#### Data format

Darwin Core Archive

#### Number of columns

29

#### Download URL

http://ipt.gbif.pt/ipt/resource?r=staphylinidae_occurrences_azores

#### Data format version

version 1.5

#### Description

The dataset was published in Global Biodiversity Information Facility platform, GBIF (Borges et al. 2021a). The following data table includes all the records for which a taxonomic identification of the species was possible. The dataset submitted to GBIF is structured as a occurrence table that has been published as a Darwin Core Archive (DwCA), which is a standardised format for sharing biodiversity data as a set of one or more data tables. The core data file contains 3903 records (occurrenceID). This IPT (Integrated Publishing Toolkit) archives the data and thus serves as the data repository. The data and resource metadata are available for download in the Portuguese GBIF Portal IPT (Borges et al. 2021a).

**Data set 2.** 

Column label	Column description
eventID	Identifier of the events, unique for the dataset.
type	Type of the record, as defined by the Public Core standard.
licence	Reference to the licence under which the record is published.
institutionID	The identity of the institution publishing the data.
institutionCode	The code of the institution publishing the data.
collectionID	The identity of the collection publishing the data.
collectionCode	The code of the collection where the specimens are conserved.
datasetName	Name of the dataset.
basisOfRecord	The nature of the data record.
occurrenceID	Identifier of the record, coded as a global unique identifier.
recordedBy	A list (concatenated and separated) of names of people, groups or organisations who performed the sampling in the field.
identifiedBy	A list (concatenated and separated) of names of people, groups or organisations who assigned the Taxon to the subject.
dateIdentified	The date on which the subject was determined as representing the Taxon.
organismQuantity	A number or enumeration value for the quantity of organisms.
organismQuantityType	The type of quantification system used for the quantity of organisms.
lifeStage	The life stage of the organisms captured.
scientificName	Complete scientific name including author and year.
scientificNameAuthorship	Name of the author of the lowest taxon rank included in the record.
kingdom	Kingdom name.
phylum	Phylum name.
class	Class name.
order	Order name.
family	Family name.
genus	Genus name.
specificEpithet	Specific epithet.
infraspecificEpithet	Infrapecific epithet.
taxonRank	Lowest taxonomic rank of the record.
establishmentMeans	The process of establishment of the species in the location, using a controlled vocabulary: in the GBIF database, we used the Borges et al. (2010) original data: 'native', 'introduced', 'endemic'.
identificationRemarks	Information about morphospecies identification (code in Dalberto Teixeira Pombo Collection).

## Additional information

We collected a total of 10,744 specimens belonging to 69 species of Staphylinidae, which represent 51% of the Staphylinidae species currently known from the Azores Archipelago (see Table 1, Suppl. material 1). Of the collected species, a total of 40 species is considered introduced (n = 5392 specimens), 25 native non-endemic (n = 5109 specimens) and four endemic (n = 188 specimens). A total of 58 individuals were identified only at genus level representing nine morphospecies and can be seen at the GBIF IPT (Borges et al. 2021a).

At archipelago level, the most abundant Staphylinidae species were the introduced *Anotylusnitidifrons* (n = 1247 specimens) and *Athetafungi* (n = 1139) and the native non-endemic *Ocypusaethiops* (n = 1219). At island level, the introduced *Anotylusnitidifrons* was also the most abundant species in Flores (n = 399) and Faial (n = 171) Islands. The introduced *Athetafungi* was the most abundant species on Graciosa (n = 303) and Santa Maria Islands (n = 649). The native *Ocypusolens* was the most abundant species on Pico Island (n = 71). The native *Ocypusaethiops* was the most abundant species on São Jorge and Terceira Islands; and the native *Quediuscurtipennis* was the most abundant species recorded on São Miguel Island (n = 327). The most abundant endemic Staphylinidae was *Notothectadryochares* (n = 119), collected in six islands (Table 1).

From this dataset, seven species are new for the Azores (Table 1; Suppl. material 1): *Aleocharafunebris* Wollaston, 1864; *Amischaforcipata* Mulsant & Rey, 1873; *Blediusunicornis* (Germar, 1825); *Carpelimustroglodytestroglodytes* (Erichson, 1840); *Cyphaseminulum* (Erichson, 1839); *Paraphloeostibagayndahensis* (MacLeay, 1871) and *Tachyporuscaucasicus* Kolenati, 1846. We also registered a total of 66 new island records (Table 1) distributed as follows across the individual islands: four for Flores, nine for Faial, six for Pico, 14 for Graciosa, nine for São Jorge, 11 for Terceira, one for São Miguel and 11 for Santa Maria.

### Updated Checklist of Azorean Staphylinidae

The latest list of Azorean rove-beetles from the Azores (Assing 2010) included a total of 115 species and subspecies of Staphylinidae. Since then, a new endemic species was recorded from Azores, *Medonvaramontis* Assing, 2013, known only from São Miguel (see Assing 2013) and the previous family Scydmaenidae (including three Azorean species) was included in the Staphylinidae as a subfamily. In addition, seven species were added in the current study (see above and Table 1). Other additions are explained below.

The catalogue of Palearctic Staphylinidae (Schülke and Smetana 2015) lists several species as recorded from the Azores that are not in the latest list of Azores species (Assing 2010). Only four of these species are now added to our updated list (see Suppl. material 1): *Aleocharaverna* Say, 1833, *Athetaaeneicollis* (Sharp, 1869), *Sepedophiluslittoreus* (Linnaeus, 1758) and *Sepedophilustestaceus* (Fabricius, 1793). The decision to add these species is based on the following reasoning:

- *Aleocharaverna*: this species was mentioned previously to Azores by Maus (1998), but without recording the island. Now we record the species for Terceira and Santa Maria (see Table 1).

-*Athetaaeneicollis*: we found no literature record. The species was added in the 2nd edition of the Staphylinidae catalogue by the Editors (Schülke and Smetana 2015), but the record is not traceable backwards. We have now found many specimens in several islands (see Table 1).

- *Sepedophiluslittoreus*: Schatzmayr (1945) Ponta Delgada (São Miguel), cited by Horion (1967), Hammond (1973) and Herman (2001), also listed in Borges (1990); the presence of this species in the Azores would be plausible.

- *Sepedophilustestaceus*: old records by Fauvel (1902) and Bernhauer (1940), citing the Crotch (1867) record of *S.sericeus*, and Méquignon (1942). All these records are doubtful and may possibly be based on misidentification, most probably *S.lusitanicus*. However, Hammond (1973), who revised the British species and described *S.lusitanicus*, explicitly wrote that he had seen *S.testaceus* from the Azores. The species was also listed by Borges (1990).

For 14 species listed in the Palaearctic Catalogue as present in the Azores (Schülke and Smetana 2015), we found no clear support for their addition to the current list of Azorean rove-beetles:

- *Athetamaderensis*: recorded by Brinck (1977). The record is based on confusion with *Athetapasadenae* (see Gusarov 2016).

- *Athetazosterae*: recorded by Bernhauer (1940), Méquignon (1942) and Israelson (1984) (misidentification). Later identified as *Athetanigra* (Kraatz).

- *Astenusgracilis*: record most likely based on Crotch 1867and Méquignon (1942); almost certainly misidentification of *A.lyonessius*.

- *Cafiussericeus*: recorded by Israelson (1984) as *Remussericeus* from Santa Maria. Israelson (1990) stated that this record was based on a misidentification of *Remuspruinosus*.

- *Euplectusafer* was recorded by Méquignon (1942) from São Miguel; this record is based on misidentification of *E.infirmus*.

- *Gyrohypnuspunctulatus*: old records of Crotch (1867) and Méquignon (1942); almost certainly belong to *G.fracticornis*.

- *Leptacinusbatychrus*: recorded by Fauvel (1897), Fauvel (1902) and Bernhauer (1940) from São Miguel. This is a misidentification of *Leptacinuspusillus* (Stephens, 1833).

-*Philonthusrufipes* : was recorded as *P.immundus* by Bernhauer (1940) from São Miguel and subsequently listed by Borges (1990). *P.immundus* is a synonym of *P.ventralis* (Gravenhorst, 1802).

- *Sepedophilusmarshami*: this record is based on Crotch (1867), who recorded *Conosomussericeus* Latreille from Flores. This species was later synonymised with *S.marshami* (Hammond 1973). The record is doubtful and may refer to *S.testaceus* or *S.lusitanicus*; most likely it refers to *S.lusitanicus*.

- For five additional species, no primary records from the Azores were found. Consequently, the respective record from the Azores in the Palearctic Catalogue (Schülke and Smetana 2015) should be deleted: *Deleasterdichrous* Gravenhorst, 1802, *Placusatachyporoides* (Waltl, 1838), *Scydmaenusrufus* Müller, P.W.J. & Kunze, 1822, *Tomoglossalaeta* Eppelsheim, 1884 and *Tomoglossaluteicornis* (Erichson, 1837).

**Figure 2.**
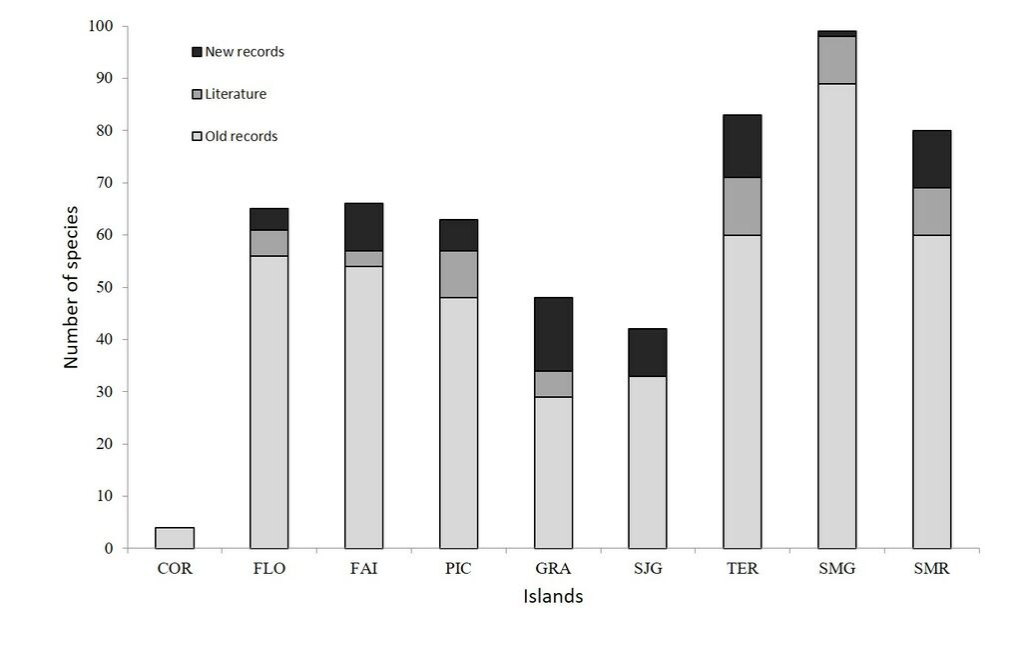
The number of rove-beetle species per island with an indication of species listed by 2010 (Old records; see Assing 2010), the species added in literature after 2010 and the new records based in the current work. COR - Corvo; FLO - Flores; FAI - Faial; PIC - Pico; SJG - São Jorge; GRA- Graciosa; TER - Terceira; SMG - São Miguel; SMR - Santa Maria.

Based on Suppl. material 1, in addition to the old records, several sources of data have contributed to the new records to Azores and individual islands (Fig. 2). Corvo and São Jorge Islands are possibly still not well studied (Fig. 2).

The current list of Azorean rove-beetles has now 136 species (Suppl. material 1). This list includes ten endemic species, but we are confident that only five are really true endemics, the other five being possibly species from other origins, but not yet recorded in the mainland. The five “true endemics” (*Athetafloresensis* Pace, 2004; *Euconnusazoricus* Franz, 1969; *Medonvaramontis* Assing, 2013; *Notothectadryochares* (Israelson, 1985) and *Phloeostibaazorica* (Fauvel, 1900)) are commonly found in native forests of Azores and, in particular, *N.dryochares* is very abundant in the canopies of Azorean endemic trees.

Most of the species assigned to native non-endemic and introduced status in Assing (2010) are mostly of Palaearctic origin and we decided to create an alternative colonisation status categorisation, assiging this species as “non-endemic” and “non-endemic cosmopolitan”. At least 16 species have a worldwide distribution and were classified as “non-endemic cosmopolitan”.

The study of Azorean Staphylinidae is far from complete. We are conducting additional surveys in the Azores (e.g. Costa and Borges 2021, Tsafack et al. 2021) and new records will be soon available covering several habitats.

## Supplementary Material

D240D015-ED6B-5089-BEF9-EC2B30D6DAD8 10.3897/BDJ.10.e78896.suppl1 Supplementary material 1 Checklist of Azorean Staphylinidae Data type Occurrences Brief description Detailed distribution of Azorean Staphylinidae in the nine Azorean islands (AZ - Azores without reference to a given island; COR - Corvo FLO - Flores; FAI - Faial; PIC - Pico; SJG - São Jorge; GRA- Graciosa; TER - Terceira; SMG - São Miguel; SMR - Santa Maria). New records per island are marked. We add also the known taxonomic or nomenclature changes in Azores in four categories (“synonym”, “different combination”, “misidentification” and “emendation/misspelling”).
“1” – Confirmed occurrence, based on Assing (2010) list; BALA - records based on BALA protocol (see Borges et al. 2016; samples part of current study); LAND-USE (see Cardoso et al. 2009, samples part of current study); MACDIV (see Malumbres-Olarte et al. 2019); ASSING - Non-standardised samples of one of us (Volker Assing) (part of current study); Marcelino - Marcelino et al. (2021); INTERF (data from project INTERFRUTA part of current study); WET – from Borges et al. (2018); AGRO – from Borges et al. (2021b); SLAM - Long term monitoring of Azorean forests using SLAM traps for Terceira Island (see Matthews et al. 2018; Borges et al. 2020; Tsafack et al. 2021); SLAM for other islands (unpublished data); ASSING 2013 (new species described in Assing 2013). File: oo_618810.txt https://binary.pensoft.net/file/618810 Paulo A. V. Borges, Volker Assing & Michael Schülke

## Figures and Tables

**Table 1.** Inventory of the Staphylinidae species collected in eight islands of Azores, from 1990 to 2015. The list includes individuals identified at species-level. Scientific name, colonisation status (CS) following the classification in Assing (2010) (int - introduced; nat - native non-endemic; end - endemic) and abundance per island are provided. Bold scientific names constitute new records for Azores and bold numbers new records for a given island. FLO - Flores; FAI - Faial; PIC - Pico; SJG - São Jorge; GRA- Graciosa; TER - Terceira; SMG - São Miguel; SMR - Santa Maria.

Scientific Name	CS	FLO	FAI	PIC	GRA	SJG	TER	SMG	SMR	Total
*Aleocharabipustulata* (Linnaeus, 1760)	int	5	1	0	3	1	63	0	11	84
*Aleocharaclavicornis* L. Redtenbacher, 1849	int	**1**	0	0	0	0	0	0	0	1
***Aleocharafunebris* Wollaston, 1864**	nat	0	0	0	**1**	0	0	0	0	1
*Aleocharapuberula* Klug, 1833	int	0	0	11	0	0	0	0	0	11
*Aleocharaverna* Say, 1833	int	0	0	0	0	0	**1**	0	6	**7**
*Aloconotasulcifrons* (Stephens, 1832)	nat	2	23	0	**1**	0	186	11	3	226
*Amischaanalis* (Gravenhorst, 1802)	int	13	31	1	0	0	244	1	21	311
***Amischaforcipata* Mulsant & Rey, 1873**	int	0	0	0	0	0	**1**	0	**18**	19
*Anotyluscomplanatus* (Erichson, 1839)	int	0	0	1	0	0	0	0	0	1
*Anotylusnitidifrons* (Wollaston, 1871)	int	399	171	**28**	**168**	**1**	246	234	0	1247
*Anotylusnitidulus* (Gravenhorst, 1802)	int	0	1	0	0	0	2	0	5	8
*Astenuslyonessius* (Joy, 1908)	nat	4	4	0	**2**	0	9	0	6	25
*Athetaaeneicollis* (Sharp, 1869)	int	0	**2**	**10**	**4**	0	**123**	2	**24**	165
*Athetaatramentaria* (Gyllenhal, 1810)	int	0	0	0	0	0	10	0	0	10
*Athetafungi* (Gravenhorst, 1806)	int	**2**	**24**	13	**303**	**5**	136	7	649	1139
*Athetanigra* (Kraatz, 1856)	int	0	0	0	0	0	1	0	0	1
*Athetapalustris* (Kiesenwetter, 1844)	int	0	0	0	**1**	1	1	1	0	4
*Athetapasadenae* Bernhauer, 1906	nat	0	0	0	0	0	338	0	0	338
***Blediusunicornis* (Germar, 1825)**	int	0	0	0	0	0	**1**	0	0	1
*Carpelimusbilineatus* (Stephens, 1834)	int	0	0	0	0	0	0	0	130	130
*Carpelimuscorticinus* (Gravenhorst, 1806)	nat	20	43	**2**	2	2	32	3	3	107
*Carpelimuspusillus* (Gravenhorst, 1802)	int	0	0	0	0	0	0	0	183	183
***Carpelimustroglodytestroglodytes* (Erichson, 1840)**	int	0	0	0	0	0	0	**1**	0	1
*Carpelimuszealandicus* (Sharp, 1900)	int	0	0	0	0	0	0	2	**3**	5
*Coproporuspulchellus* (Erichson, 1839)	int	0	0	2	3	0	210	0	3	218
*Cordaliaobscura* (Gravenhorst, 1802)	int	54	41	0	38	0	435	0	51	619
***Cyphaseminulum* (Erichson, 1839)**	int	0	0	0	**1**	0	**4**	0	0	5
*Euplectusinfirmus* Raffray, 1910	int	0	1	0	**1**	0	2	0	0	4
*Gabriusnigritulus* (Gravenhorst, 1802)	int	0	0	0	4	0	36	1	6	47
*Gyrohypnusfracticornis* (Müller, 1776)	int	0	0	4	0	0	37	0	0	41
*Habroceruscapillaricornis* (Gravenhorst, 1806)	nat	0	0	3	0	4	**3**	0	0	10
*Hydrosmectalongula* (Heer, 1839)	nat	0	0	0	0	0	0	9	0	9
*Hypomedondebilicornis* (Wollaston, 1857)	nat	0	0	0	0	0	0	0	1	1
*Lithocharisnigriceps* Kraatz, 1859	int	0	0	0	0	0	0	0	**23**	23
*Lithocharisochracea* (Gravenhorst, 1802)	int	0	0	0	0	0	0	0	7	7
*Medonapicalis* (Kraatz, 1857)	nat	0	1	0	0	0	0	0	0	1
*Medonvaramontis* Assing, 2013	end	0	0	0	0	0	0	5	0	5
*Nacaeusimpressicollis* (Motschulsky, 1858)	int	0	0	0	0	0	**2**	0	0	2
*Notothectacaprariensis* (Israelson, 1985)	end	0	0	0	0	0	0	1	0	1
*Notothectadryochares* (Israelson, 1985)	end	0	0	**5**	**1**	**1**	**48**	38	**26**	119
*Ocypusaethiops* (Waltl, 1835)	nat	0	0	0	15	**204**	1000	0	0	1219
*Ocypusolens* (Müller, 1764)	nat	34	50	71	1	202	229	35	5	627
*Oligotaparva* Kraatz, 1862	int	0	0	0	0	0	0	0	5	5
*Oligotapumilio* Kiesenwetter, 1858	nat	0	**7**	0	**1**	0	**29**	0	**5**	42
*Oxytelussculptus* Gravenhorst, 1806	int	0	0	0	0	0	0	0	26	26
***Paraphloeostibagayndahensis* (MacLeay, 1871)**	int	0	**3**	0	0	0	**200**	0	0	203
*Phacophallusparumpunctatus* (Gyllenhal, 1827)	int	0	0	0	0	0	0	0	9	9
*Philonthusdiscoideus* (Gravenhorst, 1802)	int	0	0	0	0	0	0	0	**2**	2
*Philonthusquisquiliariusquisquiliarius* (Gyllenhal, 1810)	int	0	0	0	0	0	0	48	0	48
*Philonthusumbratilis* (Gravenhorst, 1802)	int	0	0	0	0	0	0	2	0	2
*Philonthusventralis* (Gravenhorst, 1802)	nat	0	0	0	0	0	0	0	2	2
*Phloeonomuspunctipennis* Thomson, 1867	nat	0	**2**	**1**	2	1	8	0	7	21
*Phloeostibaazorica* (Fauvel, 1900)	end	0	0	3	**17**	**1**	6	36	0	63
*Platystethusnitens* (Sahlberg, 1832)	nat	2	0	3	0	0	0	0	11	16
*Proteinusatomarius* Erichson, 1840	nat	0	**24**	14	0	**3**	52	9	**17**	119
*Pseudoplectusperplexus* (Jacquelin du Val, 1854)	nat	**2**	0	0	0	0	**11**	0	0	13
*Quediuscurtipennis* Bernhauer, 1908	nat	0	**1**	0	0	**3**	**73**	327	270	674
*Quediussimplicifrons* Fairmaire, 1862	nat	0	8	14	0	59	16	3	1	101
*Rugilusorbiculatus* (Paykull, 1789)	nat	104	25	1	72	2	344	2	217	767
*Sepedophiluslusitanicus* Hammond, 1973	nat	0	1	19	19	**35**	16	93	11	194
*Stenomastaxmadeirae* Assing, 2003	nat	0	0	0	0	0	579	0	0	579
*Stenusguttulaguttula* Müller, 1821	nat	0	**4**	0	0	0	2	0	0	6
*Suniuspropinquus* (Brisout de Barneville, 1867)	nat	0	0	0	**1**	0	1	0	3	5
***Tachyporuscaucasicus* Kolenati, 1846**	int	0	0	0	0	0	0	0	**1**	1
*Tachyporuschrysomelinus* (Linnaeus, 1758)	int	379	**6**	0	12	0	4	2	32	435
*Tachyporusnitidulus* (Fabricius, 1781)	int	8	2	0	**4**	0	9	1	270	294
*Trichiusarobustula* Casey, 1893	int	**1**	0	**1**	0	**3**	4	0	**7**	16
*Trichophyapilicornis* (Gyllenhal, 1810)	nat	0	0	0	0	0	3	0	0	3
*Xantholinuslongiventris* Heer, 1839	int	0	0	2	5	0	50	0	0	57
